# Cell surface α2,3-linked sialic acid facilitates Zika virus internalization

**DOI:** 10.1080/22221751.2019.1590130

**Published:** 2019-03-22

**Authors:** Chee Wah Tan, Catherine Hong Huan Hor, Swee Sen Kwek, Han Kang Tee, I-Ching Sam, Eyleen L. K. Goh, Eng Eong Ooi, Yoke Fun Chan, Lin-Fa Wang

**Affiliations:** a Programme in Emerging Infectious Diseases, Duke-NUS Medical School, Singapore, Singapore; b Neuroscience Academic Clinical Programme, Duke-NUS Medical School, Singapore, Singapore; c Department of Medical Microbiology, Faculty of Medicine, University of Malaya, Kuala Lumpur, Malaysia

**Keywords:** Zika virus, flavivirus, sialic acid, neural progenitor cells, internalization

## Abstract

The emergence of neurotropic Zika virus (ZIKV) raised a public health emergency of global concern. ZIKV can cross the placental barrier and infect foetal brains, resulting in microcephaly, but the pathogenesis of ZIKV is poorly understood. With recent findings reporting AXL as a type I interferon antagonist rather than an entry receptor, the exact entry mechanism remains unresolved. Here we report that cell surface sialic acid plays an important role in ZIKV infection. Removal of cell surface sialic acid by neuraminidase significantly abolished ZIKV infection in Vero cells and human induced-pluripotent stem cells-derived neural progenitor cells. Furthermore, knockout of the sialic acid biosynthesis gene encoding UDP-N-acetylglucosamine-2-epimerase/N-acetylmannosamine kinase resulted in significantly less ZIKV infection of both African and Asian lineages. Huh7 cells deficient in α2,3-linked sialic acid through knockout of ST3 β-galactoside-α2,3-sialyltransferase 4 had significantly reduced ZIKV infection. Removal of membrane-bound, un-internalized virus with pronase treatment revealed the role of sialic acid in ZIKV internalization but not attachment. Sialyllactose inhibition studies showed that there is no direct interaction between sialic acid and ZIKV, implying that sialic acid could be mediating ZIKV-receptor complex internalization. Identification of α2,3-linked sialic acid as an important host factor for ZIKV internalization provides new insight into ZIKV infection and pathogenesis.

## Introduction

Zika virus (ZIKV) is an arthropod-borne RNA virus in the genus *Flavivirus* with other vector-borne viruses significant to human health, such as dengue virus (DENV), yellow fever virus (YFV), West Nile virus (WNV), and Japanese encephalitis virus (JEV) [1]. ZIKV was first isolated from a febrile sentinel rhesus macaque in 1947 and from an *Aedes africanus* mosquito in 1948 in Zika Forest, Uganda [2]. ZIKV infection has been associated with mild symptoms such as fever, rash, arthralgia, and conjunctivitis. Sporadic cases of ZIKV infections were reported over the next half century before ZIKV emerged in major outbreaks in Yap Island in 2007 [3], French Polynesia in 2013 [4], and Brazil in 2015 [5]. These ZIKV outbreaks have been associated with Guillian-Barré syndrome and congenital microcephaly [6, 7].

The *bona fide* entry receptors for flaviviruses remain unknown, and many cell surface expressed molecules could contribute to infection. These include C-type lectin DC-SIGN, L-SIGN, and phosphatidylserine receptors such as members of the T-cell Ig mucin (TIM) family and the TYRO3, AXL, and MERTK (TAM) family [8]. The TAM receptor AXL, through soluble intermediates growth arrest-specific 6 (Gas6) was recently shown to support ZIKV infection of human foreskin fibroblast [9], glial cells [10], neural stem cells [11,12], and foetal endothelial cells [13]. However, recent findings also suggest that AXL is not required in ZIKV infection in mouse models [14–16], neural progenitor cells, and cerebral organoids [17]. These contrasting findings suggested that AXL is not involved in ZIKV entry. Overall, the mechanism underlying ZIKV and/or other flaviviruses entry into host cells remains unclear.

Cell surface carbohydrates, especially heparan sulfate and sialic acid, are often utilized by viruses as attachment or entry receptors. Multiple flaviviruses, including DENV [18], WNV [19], and JEV [20], are known to use cell surface heparan sulfate as an attachment receptor. However, our previous findings suggested that heparan sulfate has no role in ZIKV infection [21]. Sialic acids are typically found on terminating branches of N-glycans, O-glycans and glycosphingolipids (gangliosides). Sialic acid may mediate virus binding and infection of cells, or alternatively can act as decoy receptors that bind virions and block virus infection [22]. Sialic acid is known to be an attachment or entry receptor for multiple viruses of significant public health concern, including human and avian influenza viruses [23,24], paramyxoviruses [25], picornaviruses [26–30], and coronaviruses [31,32]. Many sialic acid-terminated glycan binding viruses have evolved to select for specific interactions with particular sialic acid forms and linkages on different hosts and tissues, which often play important roles in the tropism of the virus [22,33].

In this study, we provide evidence that cell surface sialic acid facilitates ZIKV infection in Vero, Huh7, and induced-pluripotent stem cells (iPSC)-derived human neural progenitor cells. This result was observed across both African and Asian lineages of ZIKV.

## Materials and methods

### Cells culture

African green monkey kidney (Vero, ATCC # CCL-81), Vero clone E6 (ATCC # CRL-1586), human hepatoma (Huh7) cells, and Madin Darby canine kidney (MDCK, ATCC # CCL-34) cells were grown and maintained in Dulbecco’s modified Eagle medium (DMEM, Gibco) supplemented with 10% FBS. Mosquito *Aedes albopictus* (C6/36, ATCC # CRL-1660) cells were grown and maintained in RPMI 1640 medium (Gibco) supplemented with 10% FBS.

### Generation of human iPSC and induction of neural progenitor cells

Human iPSC was reprogrammed from human dermal fibroblasts using an episomal vector as previously described [54,55]. Briefly, the expression vectors (pCXLE-hOCT3/4-shp53, pCXLE-hUL, and pCXLE-hSK) were electroporated into fibroblast cells using Neon transfection system (Thermo Fisher Scientific) according to the manufacturer’s protocol. Electroporated cells were seeded on Matrigel-coated dishes in DMEM medium supplemented with 10% FBS and incubated at 37°C with 5% CO_2_ for 2 days. Culture medium was replaced with mTesR1 (STEMCELL Technologies) on day 3. Medium was refreshed daily until human iPSC colonies were ready for isolation.

Induction of human neural progenitor cells was performed as previously described [55]. Briefly, iPSC culture in mTesR1 was changed to neural induction medium (DMEM/F-12 medium containing neurobasal medium, N2, B27, GlutaMAX, Pen/Strep, 5 µg/ml bovine serum albumin, 10 ng/ml LIF, 4 µM CHIR99021, 3 µM SB431542, and 0.1 µM Compound E) at 20% confluency. Culture medium was refreshed every two days for 7 days and replaced with neural progenitor cells maintenance medium (DMEM/F-12 medium containing neurobasal medium, N2, B27, GlutaMAX, Pen/Strep, 5 µg/ml bovine serum albumin, 10 ng/ml LIF, 3 µM CHIR99021, 2 µM SB431542). The culture was maintained in the maintenance medium and replenished every second day for another 7 days before the first subculture. Neural progenitor cells were subsequently maintained in the maintenance medium.

### Viruses

ZIKV strains PF13/251013-18 from French Polynesia (NCBI accession no. KX369547, gift from Didier Musso at Institut Louis Malardé), MR766 from Uganda (NCBI accession no. KX377335), Paraiba_01/2015 from Brazil (NCBI accession no. KX280026, gift from Pedro F. C. Vasconcelos at Instituto Evandro Chagas) and ZKA-16-922 from National Public Health Laboratory, Ministry of Health, Singapore (NCBI accession no. MH255601) were propagated in Vero cells. PF13, Paraiba, and ZKA-16-922 are from the Asian lineage of ZIKV, while MR766 is from the African lineage. DENV-1 (EDEN2402), DENV-2 (New Guinea C strain), YFV (vaccine strain – 17D), Pteropine orthoreovirus PRV3M (commonly known as Melaka virus) [56], were also propagated in Vero cells. DENV3 (EDEN863) was propagated in C6/36 cells. Human influenza virus A (H1N1) PR8 (ATCC # VR-95) and A/NWS/33 (ATCC # VR-219) were propagated in MDCK cells in DMEM supplemented with 0.3% BSA, 25 mM HEPES and 1 µg/ml TPCK-treated trypsin. Influenza virus A/NWS/33 is a neurotropic strain derived from human strain A/WS/33 by intracerebral inoculation into mice brain [57]. Influenza virus and PRV3M were used as controls in this study.

Pseudotyped MERS coronavirus was prepared by transfection of 10 µg of pcDNA3.1-MERS-CoV Spike (EMC/2012 strain) and 10 µg of pNL4.3-EGFP lentivirus vector into HEK293 cells using FuGene 6 (Promega) in a 10 cm culture dish. Pseudotyped virus was harvested 48 h post-transfection. Pseudotyped vesicular stomatitis virus (VSV) was prepared by transfecting 10 µg of pCMV-VSV-G (Addgene) into HEK293 cells using FuGene 6 (Promega). At 24 h post-infection, pCMV-VSV-G transfected cells were infected with VSVΔG-EGFP-VSV-G seed virus for 1 h. Cells were washed twice with PBS and replenished with complete growth medium. At 48 h post-infection, the pseudotyped VSV was harvested, clarified and kept in −80°C.

### Removal of cell surface sialic acid by neuraminidase treatment

Vero cells were grown in 96-well plates and incubated with increasing concentrations of neuraminidase from *Clostridium perfringens* and *Arthrobacter ureafaciens* in serum-free DMEM for 1 h at 37°C. Cells were washed twice with serum-free DMEM followed by ZIKV infection at an MOI of 0.1 for 1 h at 37°C. The inoculum was removed and infected cells were washed twice with serum-free DMEM, and replenished with 2% FBS DMEM. Virus in the supernatant was titrated by plaque assay at 72 hpi.

### Pre-treatment of cells with lectins

Vero cells in 96-well plates were pre-incubated with increasing concentrations of WGA and ConcA lectin for 1 h at 37°C, followed by washing with serum-free DMEM. Lectin-treated cells were then infected with ZIKV at an MOI of 0.1 for 1 h at 37°C. The inoculum was removed and infected cells were washed twice with serum-free DMEM, and replenished with 2% FBS DMEM. Viruses in the supernatant were titrated by plaque assay 72 hpi.

### Plaque assays

Overnight cultured Vero cells (5 × 10^4^ cells/well) in a 24-well plate were infected with 10-fold serially diluted ZIKV or DENV for 1 h at 37°C. After incubation, the inoculum was removed and cells were immediately replenished with plaque medium supplemented with 0.8% carboxylmethylcellulose (CMC). YFV-17D was titrated in Vero, clone E6 using 0.8% CMC. ZIKV and DENV-infected cells were incubated for 5 and 4 days in a CO_2_, respectively. ZIKV and YFV-17D infected cells were fixed and stained with 4% paraformaldehyde and 0.25% crystal violet, respectively. DENV-infected cells were fixed and immunostained with mouse anti-DENV envelope antibody followed by HRP-conjugated anti-mouse antibody. The foci were developed using TrueBlue peroxidase substrate (Sera Care, USA).

For human influenza viruses, overnight cultured MDCK cells (2 × 10^5^ cells/well) in a 12-well plate were infected with serially diluted influenza viruses for 1 h at 37°C. Following incubation, the inoculum was removed and replenished with DMEM supplemented with 0.3% BSA, 25 mM HEPES, 1 µg/ml TPCK-treated trypsin and 0.8% Avicel. At 2 days post-infection, infected cells were fixed and stained with 4% paraformaldehyde and 0.25% crystal violet, respectively.

### Immunofluorescence assay

ZIKV-infected cells in an 8-well chamber slide were fixed with 4% paraformaldehyde for 10 min followed by permeabilization with 0.25% Triton-X-100 in PBS. Cells were then blocked with 1% BSA in PBS for 1 h at room temperature. ZIKV envelope antigens were immunostained with primary anti-ZIKV envelope monoclonal antibody (EastCoast Bio) at 1:1000 dilution and secondary NL-493-conjugated anti-mouse IgG antibody (R&D Systems) for 1 h at room temperature. Cell nuclei were stained with Hoechst 33342 (Sigma) for 10 min. Immunofluorescence was detected with a fluorescent microscope (Nikon).

For sialic acid staining, cells were fixed with 4% paraformaldehyde for 10 min. Biotinylated elderberry bark lectin (SNA, Vector labs), biotinylated *Maackia amurensis* lectin II (MAL-II, Vector labs) and FITC-conjugated wheat germ agglutinin lectin (WGA, Sigma) were used to stain α2,6-linked, α2,3-linked and total sialic acid, respectively. A final concentration of 20 µg/ml of the lectins was added into fixed cells for 1 h at room temperature. Streptavidin NL-493 (R&D systems) were added at 1:1000 dilution for 1 h at room temperature. The nuclei were stained with DAPI (Sigma) and the fluorescence images were captured using Cytation 5 imager (BioTek).

### Generation of sialic acid and α2,3-linked sialic acid-knockout cells using CRISPR-Cas9

Sialic acid-knockout cells were generated using the CRISPR-Cas9 gene-editing method by targeting a gene involved in sialic acid biosynthesis – UDP-N-acetylglucosamine-2-epimerase/N-acetylmannosamine kinase (GNE). Four sgRNA sequences [58] were cloned into the pSpCas9(BB)-2A-EGFP (PX458) (Addgene) plasmid as previously described by Ran et al. [59] with some modifications. Pools of four plasmids were transfected into Vero cells using TransIT-LT1 (Mirus). At 24 h post-transfection, cells were trypsinized and GFP-positive cells were sorted using FACS ARIA III. GFP-positive cells were then seeded into 96-well plates for clonal expansion. Vero cells with GNE knockout were validated by RT–PCR and FITC-conjugated WGA lectin staining.

To generate α2,3-linked sialic acid-knockout Huh7 cells, an sgRNA sequence targeting the ST3GAL4 gene was cloned into pSpCas9(BB)-2A-EGFP, and followed by transfection into Huh7 cells using Lipofectamine 3000 (Invitrogen). GFP-positive cells were sorted 24 h post-transfection, and seeded into 96-well plates for clonal expansion. The knockout cells were validated by DNA sequencing and sialic acid staining using α2,3-linked and α2,6-linked sialic acid-binding lectins.

### Construction of GNE expression vector and restoration of GNE expression in VeroΔGNE

Total RNA from Vero cells were extracted using E. Z. N. A Total RNA kit (Omega BioTek, USA) according to the manufacturer’s instructions. cDNA was synthesis was performed using gene specific primers targeting GNE mRNA using ImProm II reverse transcriptase (Promega). The GNE ORF was amplified using Q5 high-fidelity DNA polymerase (NEB) and cloned into pCAGGS expression vector at *Not*I and *Nhe*I restriction sites. The clones were validated by DNA sequencing.

VeroΔGNE cells were seeded in a 24-well plate (1 × 10^5^ cells/well). 500 ng of the pCAGGS-GNE were transfected into each well of the 24-well plate using Lipofectamine 3000 and P3000 reagents, according to the manufacturer’s instructions. Medium change was performed 4 h post-transfection and infection was performed 48 h post-transfection using ZIKV MR766 at 5 × 10^4^ PFU. At 1 h post-infection, the infected cells were replenished with either serum-free DMEM or DMEM supplemented with 2% FBS. Virus titres were determined at 48 hpi by plaque assay.

### Virus attachment and internalization analysis using pronase treatment

Neuraminidase-treated or untreated Vero cells, and VeroΔGNE cells were infected with ZIKV PF13 at an MOI of 1 for 2 h at 37°C. Infected cells were washed three times with Dulbecco’s PBS (DPBS), followed by pronase (Roche) treatment at a final concentration of 1 mg/ml in DPBS for 10 min at room temperature. Pronase-treated cells were washed five times with DPBS and the total RNA was extracted using E. Z. N. A Total RNA kit I (Omega BioTek).

ZIKV and H1N1 genomic RNA and housekeeping (small nuclear ribonucleoprotein D3, SNRPD3) mRNA levels were determined by real-time PCR. In brief, cDNA was synthesized using the Quantitect Reverse Transcription kit (Qiagen) and real-time PCR was performed with CFX96 real-time PCR detection system (Bio-Rad) using SensiFast SYBR No-ROX kit (Bioline).

### Statistical analysis

Statistical details of experiments are found in the corresponding figure legends. Viral titres in treated and untreated cells were analysed by the Student’s *t* test. Statistical analyses were conducted with GraphPad Prism 8 (GraphPad). *P* values of < 0.05 were considered statistically significant.

## Results

### Removal of surface sialic acid from Vero cells by neuraminidase significantly reduced ZIKV infection

**Figure 1.**
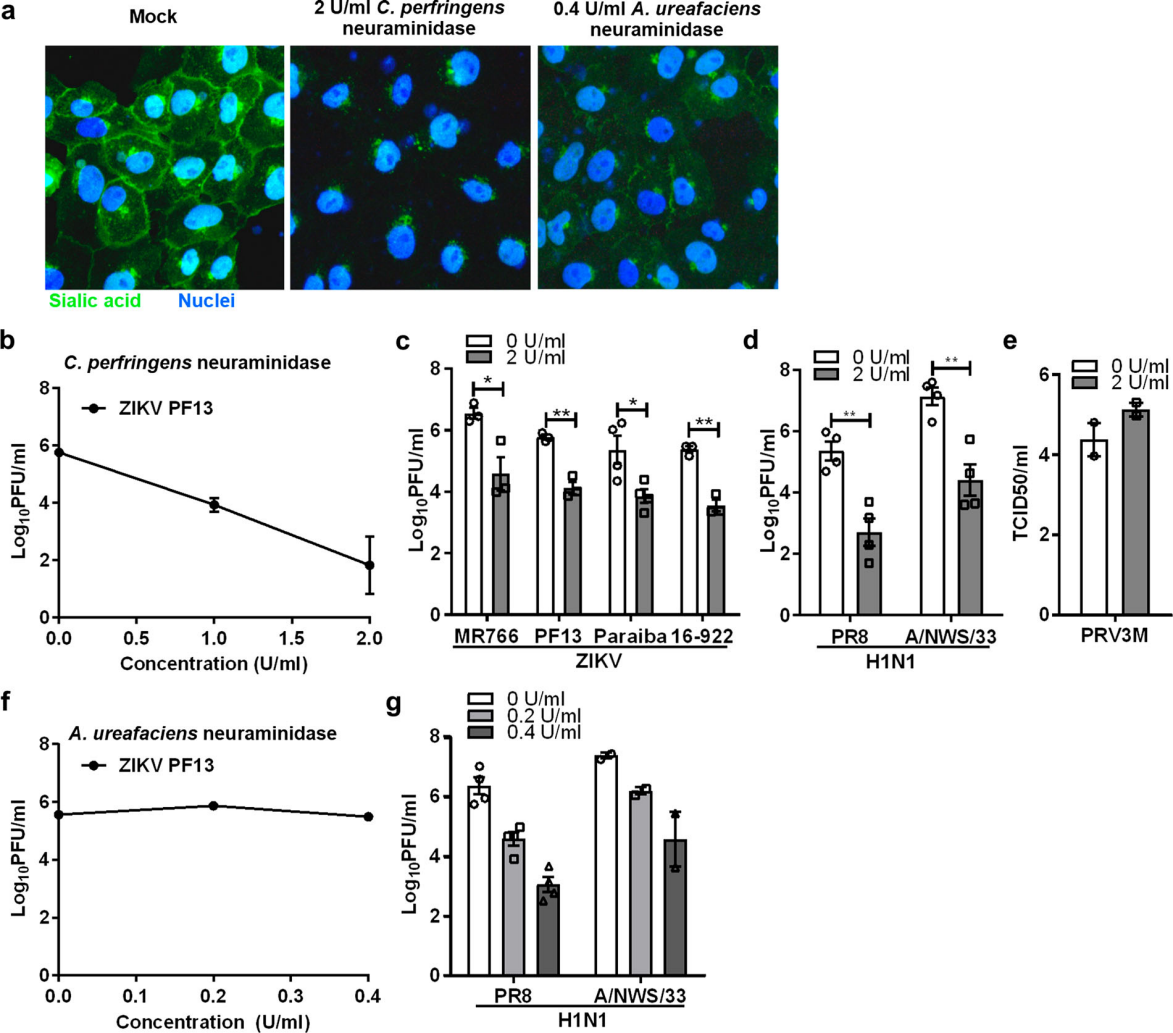
Role of sialic acid in Vero cells during ZIKV infection. (**a**) Sialic acid staining of Vero and neuraminidases-treated cells were performed using FITC-conjugated WGA lectin for 10 min at 37°C. Cell nuclei were stained with Hoechst 33342. (**b)** Vero cells were pre-treated with increasing concentrations of neuraminidase from *C. perfringens* for an hour prior to ZIKV PF13 infection at a MOI of 0.1. The effect of *C. perfringens* neuraminidase-treated Vero cells on (**c**) multiple ZIKV, (**d**) H1N1 and (**e**) PRV3M infections at a final concentration of 2U/ml. The effect of *A. ureafaciens* neuraminidase that removes α2,6-linked sialic acid on (**f**) ZIKV PF13 and (**g**) H1N1 infection. Virus titres for ZIKV, DENV, and H1N1 were determined 72 hpi by plaque assay. All experiments were repeated at least two times with duplicate samples. Asterisks indicate statistically significant differences (*P < .05; **P < .01; ***P < .001). Error bars represent means ± standard error.

Our previous findings suggested that ZIKV does not use cell surface heparan sulfate as an attachment receptor [21]. To test the role of sialic acid in ZIKV infection, we used neuraminidase to remove cell surface sialic acid prior to ZIKV infection. We first validated the efficacies of the neuraminidases in removing sialic acid using FITC-conjugated WGA lectin staining. Neuraminidase from *C. perfringens* (preferentially removing α2,3-linked sialic acid) was shown to be more efficient than neuraminidase from *A. ureafaciens* (preferentially removing α2,6-linked sialic acid) in Vero cells (Figure 1(a)).

Subsequently, it was shown that Vero cells pre-treated with increasing concentrations of neuraminidase from *C. perfringens* for 1 hr at 37°C significantly reduced ZIKV infection in a dose-dependent manner (Figure 1(b)). As shown in Figure 1(c), removal of cell surface sialic acid had the same inhibitory effect for infection of both the African (Uganda-MR766) and Asian (Brazil-Paraiba, French polynesia-PF13 and Singapore-16-922) lineages. As a positive control, Vero cells treated with *C. perfringens* neuraminidase significantly reduced infection with both human influenza viruses H1N1 PR8 and A/NWS/33 (Figure 1(d)). *C. perfringens* neuraminidase-treated Vero cells remain susceptible to PRV3M (commonly known as Melaka orthoreovirus) infection (Figure 1(e)). However, treating Vero cells with *A. ureafaciens* neuraminidase had no impact on ZIKV infection (Figure 1(f)), but reduced the infection of human influenza viruses that preferentially use α2,6-linked sialic acid as a receptor, by 3.3 and 2.8 log PFU/ml for H1N1 PR8 and A/NWS/33 at 0.4 U/ml, respectively (Figure 1(g)). This indicated that α2,6-linked sialic acid does not play a significant role during ZIKV infection.

**Figure 2.**
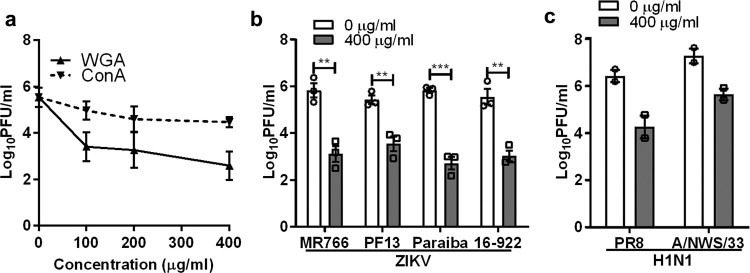
Inhibitory effect of sialic acid-binding lectin against ZIKV infection. (**a**) Vero cells were pre-treated with increasing concentration of lectin from WGA and Concanavalin A (ConA) for 1 h at 37°C followed by ZIKV PF13 infection at a MOI of 0.1. Effect of WGA lectin-treated Vero cells on multiple (**b**) ZIKV and (**c**) H1N1 strains infection. All experiments were repeated at least two times with duplicate samples. Asterisks indicate statistically significant differences (*P < .05; **P < .01; ***P < .001). Error bars represent means ± standard error.

To further confirm the role of sialic acid in ZIKV infection, Vero cells were pre-treated with a sialic acid-binding lectin, WGA, prior to ZIKV infection. An α-D-mannose binding lectin, concanavalin A (ConA) was used as control. WGA exhibited significant inhibitory activity in a dose-dependent manner, with a 2.9 log PFU/ml reduction at 400 µg/ml (Figure 2(a)). This inhibitory effect was also observed for all ZIKV strains tested (Figure 2(b)). As expected, WGA treatment inhibited both H1N1 PR8 and A/NWS/33 infections (Figure 2(c)).

### Removal of sialic acid on human iPSC-derived neural progenitor cells significantly inhibits ZIKV infection

**Figure 3.**
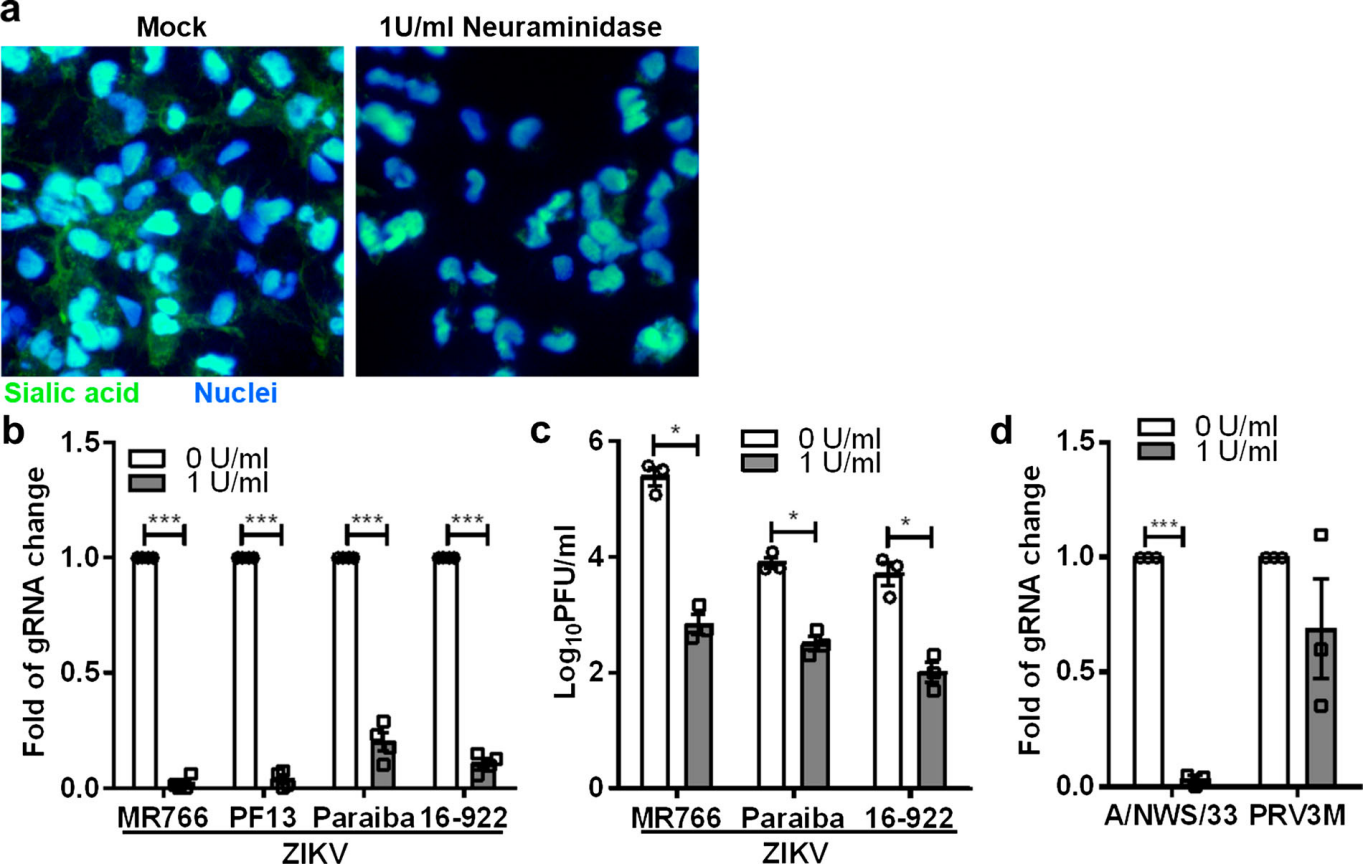
Role of sialic acid in neural progenitor cells in ZIKV infection. (**a**) Neuraminidase-treated or untreated neural progenitor cell surface sialic acids were visualized by FITC-conjugated WGA lectin staining. Hoechst 33342 were used to stain cell nuclei. Neural progenitor cells were pre-treated with 1U/ml of *C. perfringens* neuraminidase for 1 h at 37°C followed by ZIKV (MR766, PF13, Paraiba and 16-922), A/NWS/33, and PRV3M infection at a MOI of 0.1. The effect of sialic acid removal on virus infections were determined by (**b**) real-time PCR and (**c**) plaque assays. (**d**) The effect of sialic acid removal on A/NWS/33 and PRV3M were determined by real-time PCR at 72 hpi. Fold changes of viral gRNA were determined using 2^−ΔΔCT^ method after normalization with a housekeeping gene, SNRPD3. All experiments were repeated at least three times with duplicate samples. Asterisks indicate statistically significant differences (*P < .05; **P < .01; ***P < .001). Error bars represent means ± standard error.

ZIKV is known to cause microcephaly in mice and humans, likely due to its ability to disrupt neural progenitor development [34,35]. Specifically, ZIKV, MR766 was reported to efficiently infect hNPCs derived from induced-pluripotent stem cells (iPSC) [36]. Therefore, we next sought to examine whether sialic acid is also involved in ZIKV infection in human iPSC-derived NPCs. Human induced-pluripotent stem cells (iPSC), reprogrammed from human dermal fibroblasts were used for neural progenitor cell induction. As shown in Figure 3(a), neural progenitor cells treated with 1 U/ml of *C. perfringens* neuraminidase displayed less sialic acid on the cell surface. When treated at the final concentration of 1U/ml, there was a significant decrease in ZIKV infection for multiple strains as determined by real-time PCR (Figure 3(b)). Plaque assays (Figure 3(c)) showed decreasing viral titres of 2.6 log PFU/ml, 1.4 log PFU/ml and 1.7 log PFU/ml for ZIKV MR766, Paraiba and 16-922, respectively. As shown in Figure 3(c), ZIKV MR766 replicates more efficiently in neural progenitor cells as compared to other ZIKV strains. As an additional control, we demonstrate that neural progenitor cells are permissive to H1N1 A/NWS/33 and PRV3M infection. Neuraminidase treatment significantly inhibits A/NWS/33 but failed to reduce PRV3M infection (Figure 3(d)).

### Sialic acid-knockout Vero cells were less susceptible to infection by different ZIKV strains

**Figure 4.**
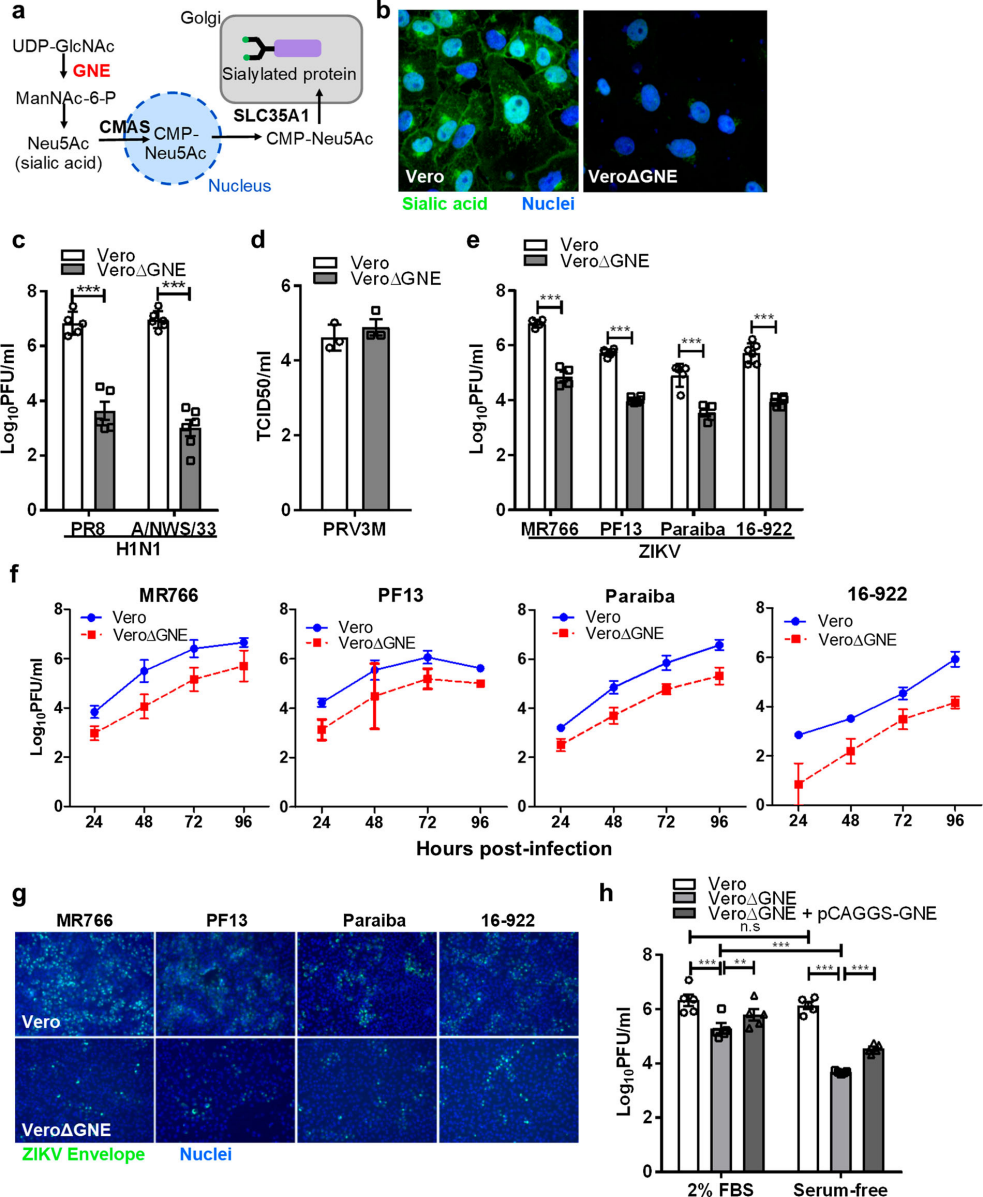
Sialic acid-knockout Vero cells are less susceptible to ZIKV infection. (**a**) Simplified schematic of sialic acid biosynthesis pathway. GNE, the gene selected for CRISPR-Cas9 knockout, is highlighted in red. N-acetylglucosamine (GlcNAc), N-acetylmannosamine (ManNAc), N-acetylglucosamine (GlcNAc), uridine diphosphate (UDP), and cytidine monophosphate (CMP). (**b**) The knockout cells were validated with sialic acid staining with FITC-conjugated WGA lectin. Cell nuclei were stained with Hoechst 33342. The susceptibility of Vero and VeroΔGNE cells to different viruses is shown: (**c**) H1N1 (PR8 and A/NWS/33), (**d**) PRV3M, (**e**) ZIKV (PF13, MR766, Paraiba and 16-922). (**f**) The replication kinetics of ZIKV in Vero and VeroΔGNE cells. (**g**) Immunofluorescence assay of ZIKV-infected Vero and VeroΔGNE cells at 48 hpi. ZIKV envelope protein was immunostained with anti-ZIKV envelope monoclonal antibody followed by NL493 anti-mouse IgG antibody. Cell nuclei were stained with Hoechst 33342. (**h**) VeroΔGNE cells transfected with pCAGGS-GNE were more susceptible to ZIKV infection. Virus titres for ZIKV and H1N1 were determined 72 hpi by plaque assay. All experiments were repeated at least three times with duplicate samples. Asterisks indicate statistically significant differences (*P < .05; **P < .01; ***P < .001). Error bars represent means ± standard error.

To generate sialic acid-knockout cells, we used the CRISPR-Cas9 gene-editing tool to delete the GNE gene. GNE encodes a bifunctional enzyme that initiates and regulates the biosynthesis of N-acetylneuraminic acid (Neu5Ac), a precursor of sialic acid (Figure 4(a)). Lectin staining showed the absence of sialic acid on the VeroΔGNE cell surface (Figure 4(b)). Functional validation was conducted by infecting wild-type and knockout Vero cell lines with influenza viruses. VeroΔGNE cells were less susceptible to both H1N1 PR8 and A/NWS/33 influenza viruses (Figure 4(c)), and showed no significant reduction in PRV3M output (Figure 4(d)).

VeroΔGNE cells remain susceptible to ZIKV infection, but with a significant reduction of infection efficiency, decreasing the titres by ∼1.4–2.2 log PFU/ml across different ZIKV strains (Figure 4(e)). Replication kinetics of ZIKV in Vero and VeroΔGNE cells imply that sialic acid is necessary for efficient ZIKV infection (Figure 4(f)). As shown in Figure 4(g), reduced levels of ZIKV envelope proteins were detected in VeroΔGNE cells compared to the wild-type Vero cells using immunofluorescence assay. Furthermore, ZIKV infection of VeroΔGNE cells yielded smaller plaque morphology as compared to the Vero cells (Fig. S1), implying that sialic acid-knockout cells restrict ZIKV infection. VeroΔGNE cells transfected with pCAGGS-GNE partially restored ZIKV (Figure 4(h)) and H1N1 infection (data not shown). This observation was even more obvious when the exogenous source of sialic acid was depleted by using FBS-free medium (Figure 4(h)).

### Cell surface α2,3-linked sialic acid facilitates ZIKV infection

**Figure 5.**
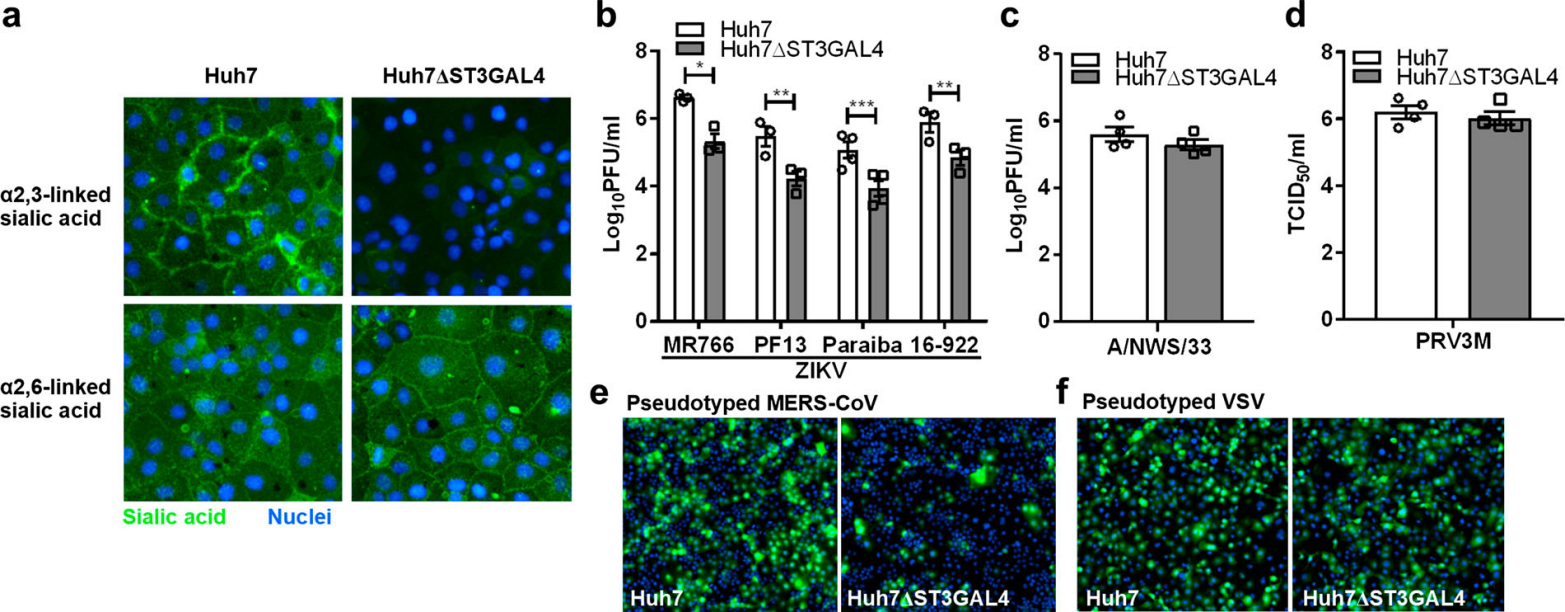
α2,3-linked sialic acid-deficient cells are less susceptible to ZIKV infection. (**a**) Sialic acid staining of huh7 and huh7ΔST3GAL4 cells. The α2,3-linked and α2,6-linked sialic acid are stained with MAL-II and SNA lectins, respectively. Cell nuclei were stained with DAPI. The susceptibility of huh7 and huhΔST3GAL4 cells to different viruses are shown: (**b**) ZIKV (MR766, PF13, Paraiba and 16-922), (**c**) H1N1 A/NWS/33, (**d**) PRV3M, (**e**) pseudotyped MERS coronavirus and (**f**) pseudotyped VSV stably expressed enhanced GFP. Cell nuclei were stained with DAPI. Fluorescent images were captured using Cytation 5 imager (BioTek). Virus titres for ZIKV and H1N1 were determined 48 hpi by plaque assay. Virus titres for PRV3M were determined 24 hpi by end-point dilution. All experiments were repeated at least three times with duplicate samples. Asterisks indicate statistically significant differences (*P < .05; **P < .01; ***P < .001). Error bars represent means ± standard error.

As demonstrated previously, removal of α2,6-linked sialic acid by *A. ureafaciens* neuraminidase failed to reduce ZIKV infection. A recent study has demonstrated that GNE-deficient cells produced distinct N-linked glycan structures with increased branching and extended poly-N-acetyllactosamine [37]. These modifications may have an impact on virus infection. To further corroborate our findings, we specifically generated α2,3-linked sialic acid-deficient Huh7 cells by knocking out ST β-galactoside-α2,3-sialyltransferase (ST3GAL4) gene using the CRISPR-Cas9 system. Huh7ΔST3GAL4 cells were deficient in α2,3-linked sialic acid but expressed normal levels of α2,6-linked sialic acid (Figure 5(a)). As demonstrated in Figure 5(b), huh7ΔST3GAL4 cells were significantly less susceptible to ZIKV infection. Deficiency of α2,3-linked sialic acid failed to reduce H1N1 A/NWS/33 and PRV3M infection (Figure 5(c,d)). Huh7ΔST3GAL4 cells were less susceptible to pseudotyped Middle East Respiratory Syndrome (MERS) coronavirus known to use α2,3-linked sialic acid [31] (Figure 5(e)). No difference in susceptibility between Huh7 and Huh7ΔST3GAL4 for pseudotyped VSV was observed (Figure 5(f)). This data further corroborate the role of α2,3-linked sialic acid during ZIKV infection.

### Cell surface sialic acid facilitates ZIKV internalization, not attachment

**Figure 6.**
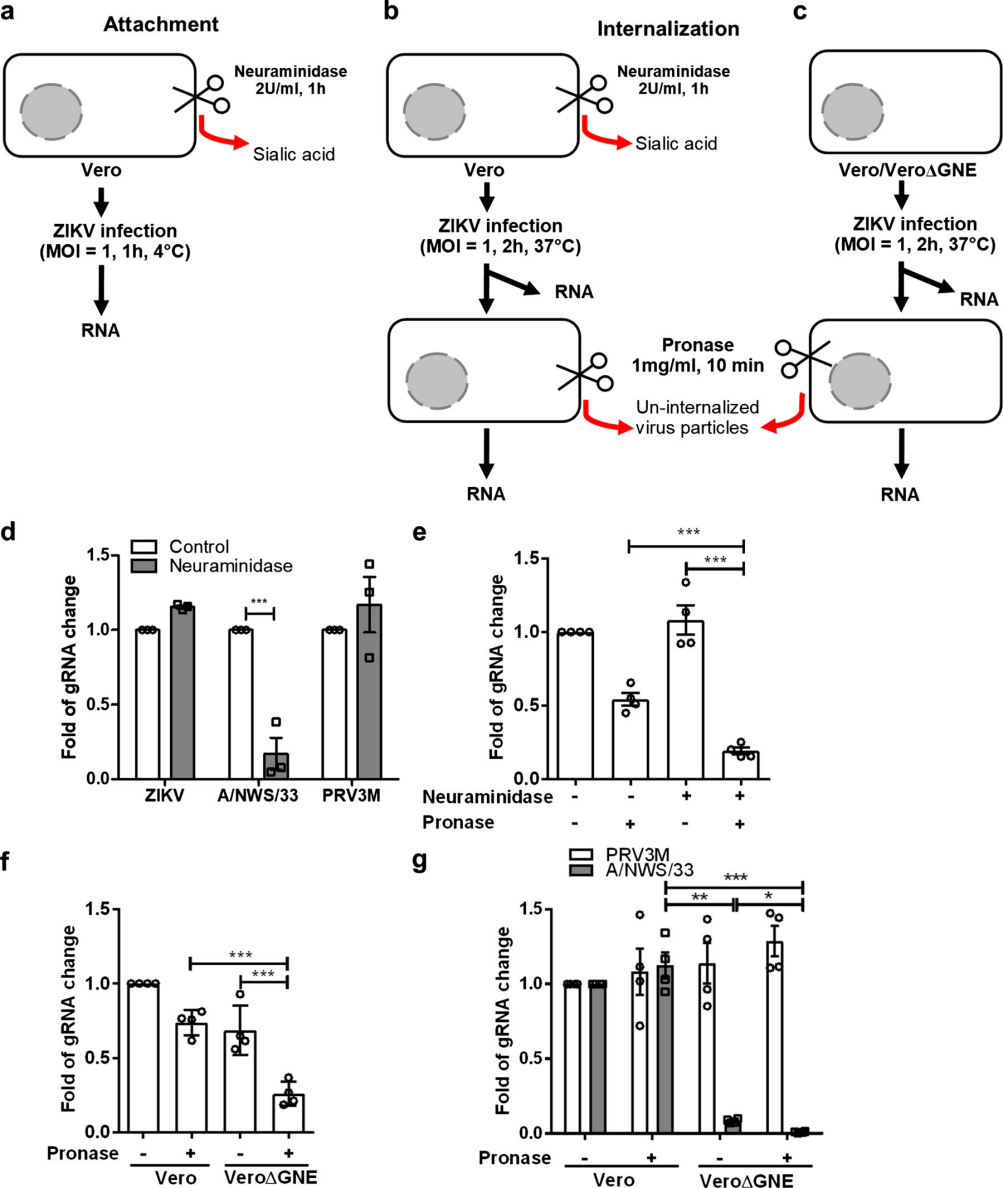
Sialic acid is involved in ZIKV internalization. Schematic illustration of the experimental design to characterize ZIKV attachment and internalization under different conditions, (**a**) attachment on neuraminidase-treated cells at 4°C, (**b**) effect of neuraminidase treatment on ZIKV internalization at 37°C and (**c**) effect of GNE knockout on attachment/internalization. Pronase (at final concentration of 1 mg/ml in DPBS) were used to remove plasma membrane-bound but un-internalized virus particle after 2 h of infection. Fold changes of viral gRNA were determined using 2^−ΔΔCT^ method after normalization with a housekeeping gene, SNRPD3. (**d**) Attachment of ZIKV, H1N1 A/NWS/33 and PRV3M on neuraminidase-treated cells at 4°C. Analysis on ZIKV internalization in (**e**) neuraminidase-treated cells and untreated cells by pronase treatment; and (**f**) VeroΔGNE and Vero cells. (**g**) Effect of GNE knockout on attachment and/or internalization of H1N1 A/NWS/33 and PRV3M. . All experiments were repeated at least three times. Asterisks indicate statistically significant differences (*P < .05; **P < .01; ***P < .001). Error bars represent means ± standard error.

Sialic acid is known to serve as an attachment or entry receptor for multiple viruses. To characterize the mechanistic role of sialic acid during ZIKV infection, neuraminidase-treated Vero or VeroΔGNE cells were incubated with ZIKV PF13 either at 4°C or 37°C to promote virus attachment and internalization, respectively. For virus internalization, ZIKV-infected cells were subjected to pronase treatment to remove plasma membrane-bound but un-internalized viral particles after incubation at 37°C (Figure 6(a,b,c)). This experiment was performed in order to differentiate the role of sialic acid in attachment versus internalization. As shown in Figure 6(d), there was no difference in ZIKV particles that attached to the neuraminidase-treated and untreated cells at 4°C. As a control, it was evident that neuraminidase-treated cells significantly reduced influenza H1N1 A/NWS/33 attachment (Figure 6(d)). By using pronase to remove un-internalized virus particles, we observed a significant reduction (3-fold) of the viral RNA in the neuraminidase-treated Vero cells (Figure 6(e)). This finding indicates that ZIKV internalization is, at least in part, dependent on sialic acid.

Besides using neuraminidase-treated cells to assess the role of sialic acid in ZIKV internalization, we also turned to our VeroΔGNE cells. Consistent with neuraminidase-treated Vero cells, VeroΔGNE cells also internalized significantly less ZIKV virus particles (Figure 6(f)). However, it is interesting to note that GNE knockout also had some effect on ZIKV attachment. It is known that GNE knockout not only stop sialic acid production, it also affects the synthesis of other carbohydrates [37]. Using the Huh7ΔST3GAL4, we further demonstrated that remove of α2,3-linked sialic acid alone had no effect on ZIKV attachment (Fig. S2). As a control, influenza H1N1 A/NWS/33 internalization was shown to be significantly reduced in VeroΔGNE (Figure 6(g)). Pronase treatment on VeroΔGNE cells has no impact on PRV3M internalization (Figure 6(g)). To characterize the sialic acid-binding activity of ZIKV, ZIKV was pre-incubated with 3′ sialyllactose and 6′ sialyllactose prior to infection of Vero cells. None of these treatments showed significant inhibition of ZIKV MR766 infection of Vero cells, while inhibition of H1N1 A/NWS/33 was observed, as expected (Fig. S3). As a control, α-mannose binding lectin, conA inhibits both ZIKV MR766 and H1N1 A/NWS/33 infection (Fig. S3). Taken together, these data suggest that there is no direct interaction between sialic acid and ZIKV, further supporting our previous finding that sialic acid is not involved in ZIKV attachment.

### Cell surface sialic acid facilitates DENV and YFV infection

**Figure 7.**
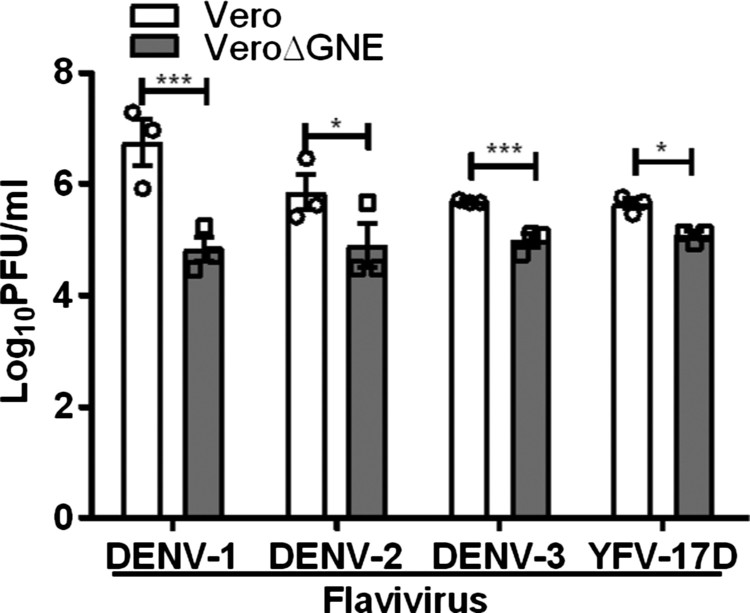
Role of sialic acid in DENV and YFV-17D infection. Vero and VeroΔGNE cells were infected with DENV-1, DENV-2, DENV-3, and YFV-17D. Virus titres were determined 72 hpi by plaque assay. All experiments were repeated three times with duplicate samples. Asterisks indicate statistically significant differences (*P < .05; **P < .01; ***P < .001). Error bars represent means ± standard error.

Next, we investigated the role of sialic acid in other flavivirus infections. Results were confirmed with sialic acid-deficient Vero cells, with 1.9 log PFU/ml and 1.1 log PFU/ml reduction for DENV-1 and DENV-2, respectively (Figure 7). Sialic acid-deficient Vero cells were also less susceptible to DENV-3 and YFV-17D infection, showing less than a log PFU/ml reduction (Figure 7).

## Discussion

Cell surface carbohydrates, including heparan sulfate and sialic acid, are often utilized by viruses as attachment and entry receptors. Sialic acids are a highly diverse family of monosaccharides that serve as terminal residues of N- and O-linked glycoproteins, glycolipids and are also components of various polysaccharides. Sialic acids are abundantly displayed on the surfaces of vertebrate cells, particularly on all mucosal surfaces. However, the role of sialic acid in flavivirus infection, in particular, ZIKV, is unknown. Multiple studies have reported that neuraminidase treatment of *Aedes albopictus* C6/36 cells failed to inhibit DENV-1, DENV-4, and WNV infection [38-40]. This could be due to the fact that insect cells are deficient in sialic acid biosynthesis enzymes and thus lack cell surface sialic acids [41–43].

In the present study, we have demonstrated that sialic acid plays an important role in ZIKV infection. Cell surface α2,3-linked sialic acid facilitates infection of ZIKV strains from both the African (Uganda) and Asia (French Polynesia, Brazil, and Singapore isolates) lineages. By using pronase to remove plasma membrane-bound but un-internalized virus particles [44], our data demonstrated that sialic acid is involved in ZIKV internalization, not attachment. This finding could explain why neuraminidase-treated HepG2 and Vero cells failed to reduce S^35^-labelled DENV-1 binding in a previous study [45]. Similarly, sialic acid is previously reported to enhance adeno-associated virus 2.5 T internalization through sialic acid-dependent endocytosis [46]. The failure of blocking ZIKV infection by pre-incubation of ZIKV with 3′ or 6′ sialyllactose and fetuin further suggests that there was no direct interaction between sialic acid and ZIKV virus particles during virus attachment. The mechanism underlying sialic acid-dependent internalization remains unclear. Sialic acids are not able to transmit signals across the plasma membrane and therefore, require additional signalling receptors to trigger internalization or endocytosis [47]. The binding of influenza A virus to sialic acid on the cells results in clustering of lipid rafts and activation of epidermal growth factor receptor and other receptor tyrosine kinases, which subsequently recruit PI3K to trigger the endocytosis pathway [47].

It is known that sialic acid can be recycled from the internalized exogenous sialylated glycoprotein present in FBS by sialin (SLC17A5). Sialin, mainly found in lysosomes, is an anion transporter capable of transporting glucuronic acid and free sialic acid out of the lysosome after it is cleaved from sialoglycoconjugates [48]. When the exogenous source of sialic acid was depleted by using FBS-free medium, the VeroΔGNE cells were significantly less susceptible to ZIKV infection. In contrast, no significant susceptibility change was observed for the wild-type Vero cells cultured in 2% FBS versus serum-free medium. This implied that VeroΔGNE cells were still able to display a low level of sialic acid on the surface when grown in the FBS-containing medium. This further highlights the important role of sialic acid in ZIKV infection. Transient expression of GNE gene is able to restore ZIKV infectivity in VeroΔGNE.

ZIKV exhibits a broad tissue tropism and persistence in body tissues and fluids, including brain, placenta, eye, and testes [49]. Initial studies suggested that ZIKV preferentially targets neural progenitor cells, providing an explanation for the abnormal developmental phenotypes observed in some pregnancies [36]. Furthermore, ZIKV replicates efficiently in neural progenitor cells, leading to a cell-cycle arrest, defects in differentiation and resulting microcephaly in a mouse model [34]. However, genetic ablation of AXL has no effect on ZIKV entry or ZIKV-mediated cell death in human iPSC-neural progenitor cells or cerebral organoids [17]. A recent study further suggests that AXL promotes ZIKV infection in astrocytes by antagonizing type I interferon signalling, rather than promoting ZIKV entry [50]. Here, we demonstrate that sialic acid is required for ZIKV infection in neural progenitor cells.

Our data suggest that ZIKV and, most likely other flaviviruses, use α2,3-linked sialic acid as an internalization factor. The exact mechanism underlying sialic acid-mediated internalization requires further investigation. To date, multiple receptors have been reported for flaviviruses [51]. It is, therefore, possible that sialic acid is not the only internalization factor used by ZIKV. Our findings may also explain why the GNE gene was not identified in the CRISPR-cas9 library screen reported by Savidis et al. [52]. GNE knockout in mice causes early embryonic lethality [53], which limits the *in vivo* investigation of the role of sialic acid in ZIKV infection in mice.

## Supplementary Material

Supplemental Material
