# Supplementary material for: Cell surface α2,3-linked sialic acid facilitates Zika virus internalization
Source: Emerg Microbes Infect. 2019 Mar 22;8(1):426–37. doi: 10.1080/22221751.2019.1590130 (PMC6455136; doi:10.1080/22221751.2019.1590130)
Supplement: Supplemental Material [file TEMI_A_1590130_SM5735.docx]

**Vero**

**Vero**

**Δ**

**GNE**

**ZIKV MR766**

**10**

**-**

**fold serial dilution**

**Vero**

**Vero**

**Δ**

**GNE**

**H1N1 A/NWS/33**


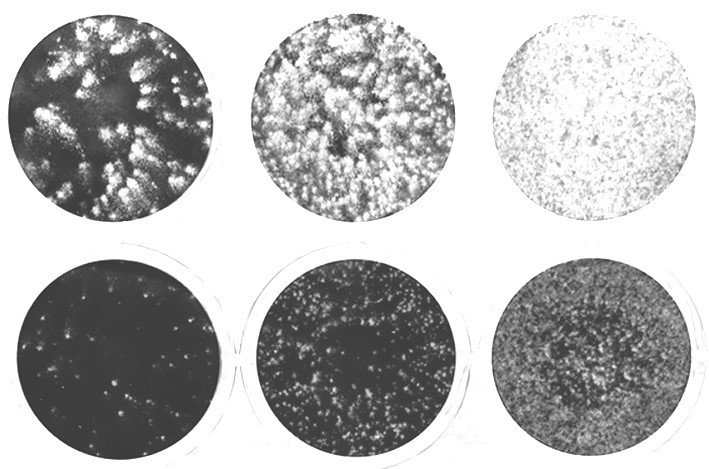

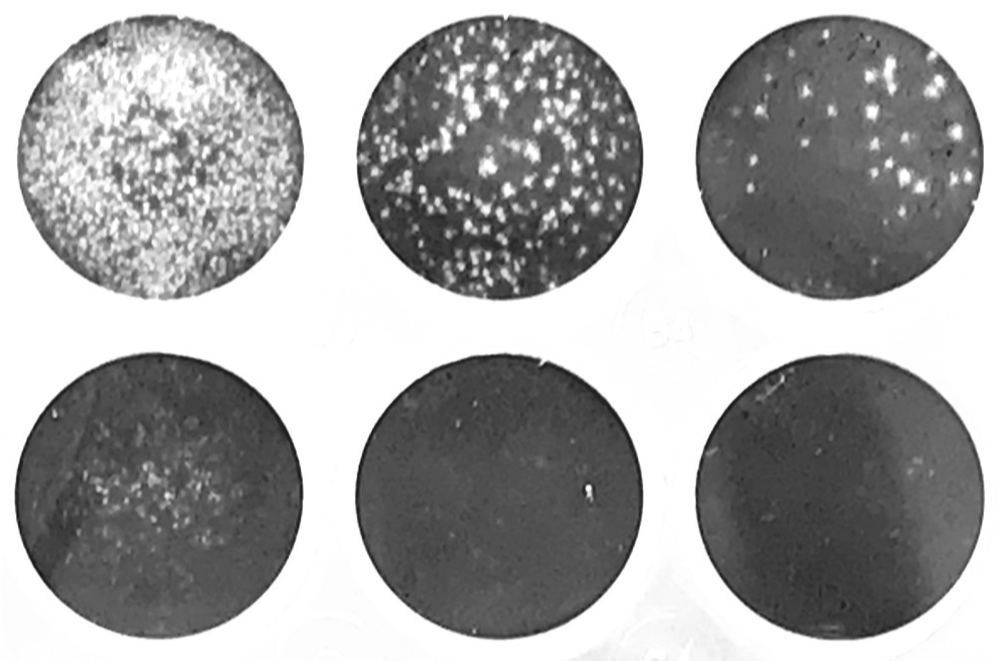


**Fig. S1.** **Plaque morphology of ZIKV MR766 and H1N1 A/NWS/33 in Vero and VeroΔGNE.** Vero and VeroΔGNE were seeded in a 12-well plate at 4 x 10^5^ cells/well in serum-free DMEM. Prior to infection, the cells were washed twice with serum-free DMEM followed by serial-diluted ZIKV MR766 and H1N1 A/NWS/33 infection. One-hour post-infection, the inoculum was removed and replenished with DMEM supplemented with 2% FBS and 0.8% CMC for ZIKV or DMEM supplemented with 0.3% BSA, 25mM HEPES, 1 µg/ml TPCK-trypsin and 0.8% Avicel for H1N1. Infected cells were fixed and stained with 4% paraformaldehyde and 0.5% crystal violet, respectively.


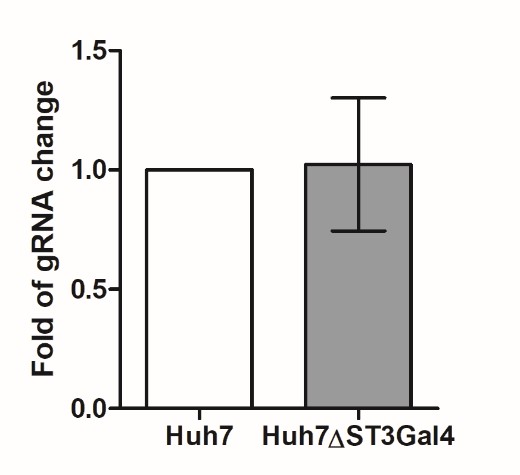


**Fig. S2. ZIKV attachment assay.** Huh7 and huh7ΔST3Gal4 cells were pre-incubated with ZIKV particle at a MOI of 1 for 1 hour at 4°C. Cells were washed 3 times with ice-cold DPBS, and the total RNA was extracted for real-time PCR analysis. Fold changes of viral gRNA were determined using 2^-ΔΔCT^ method after normalization with a housekeeping gene, SNRPD3. All experiments were repeated at least three times. Error bars represent means ± standard error.

1. **ZIKV (MR766) H1N1 (A/NWS/33)**

**3’ SL 6’SL 3’SL 6’SL**


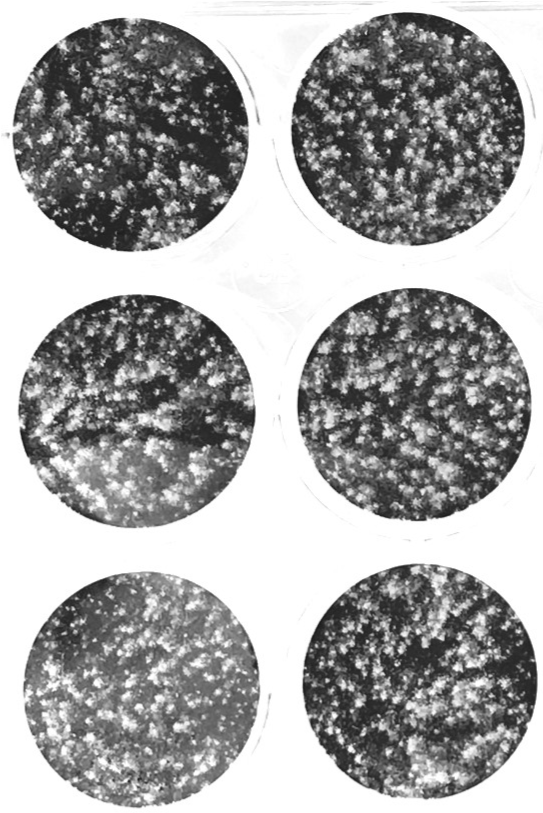

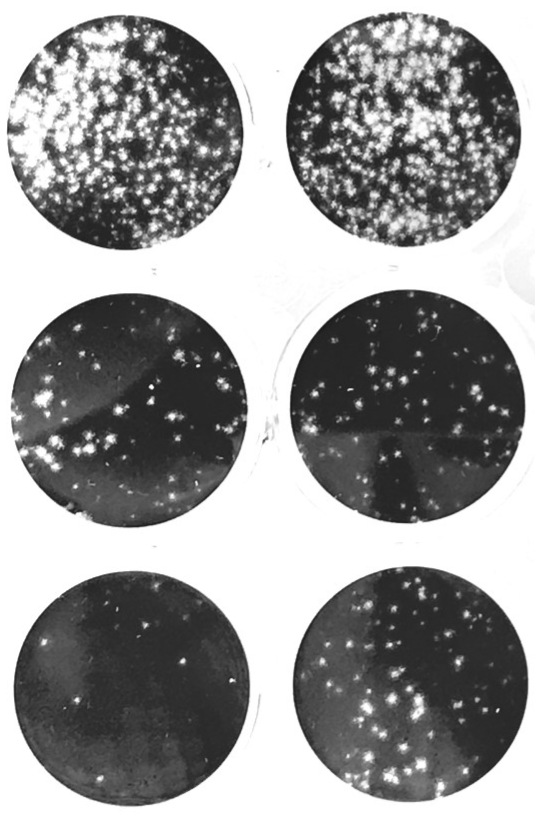


**0**

**mM**

**20**

**mM**

**40**

**mM**

1. **ZIKV (MR766) H1N1 (A/NWS/33)**

**Fetuin ConA Fetuin ConA**


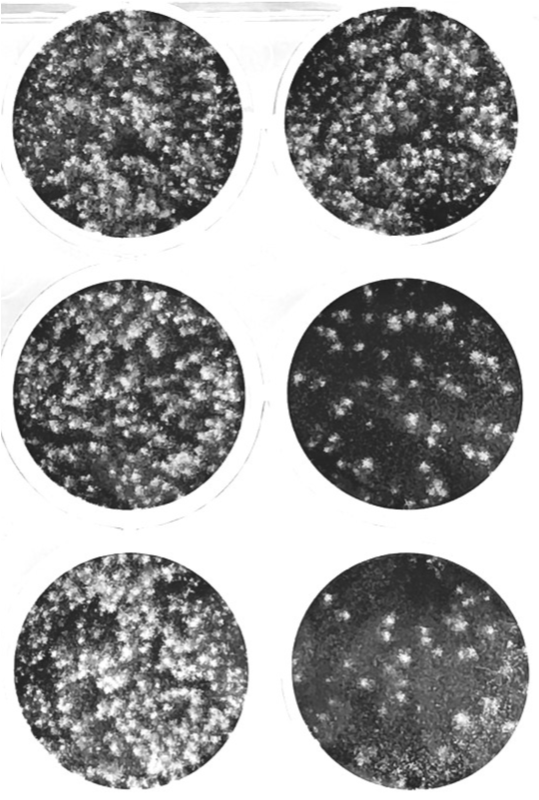

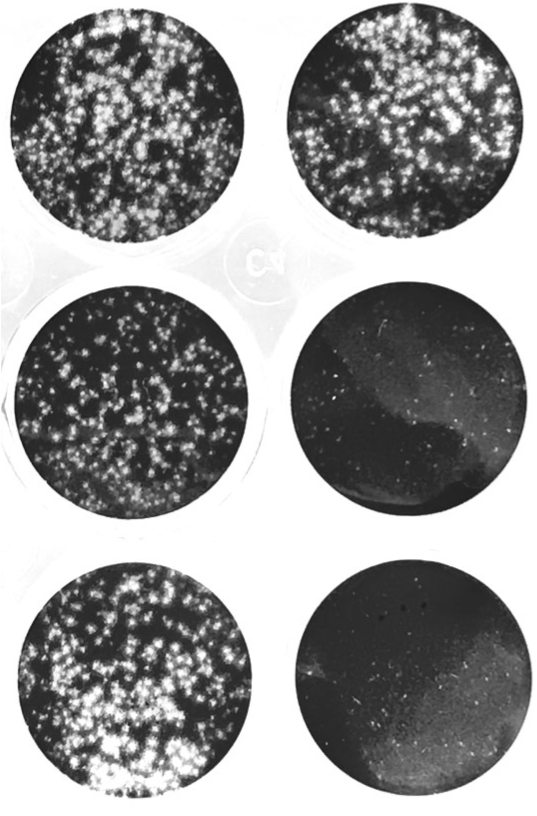


**0**

**µg/ml**

**200**

**µg/ml**

**400**

**µg/ml**

**Fig. S3. Plaque inhibition of 3’ sialyllactose, 6’ sialyllactose, fetuin and conA against ZIKV and H1N1.** Approximately of 200 PFU of ZIKV (Uganda-MR766) and H1N1 (A/NWS/33) were preincubated with increased concentration of (A) 3’ and 6’ sialyllactose (SL); (B) fetuin and conA for 1 h at 37°C. The virus-inhibitors mixed were then added into monolayer Vero cells for 1 h at 37°C. Onehour post-infection, the inoculum was removed and replenished with DMEM supplemented with 2%

FBS and 0.8% CMC for ZIKV or DMEM supplemented with 0.3% BSA, 25mM HEPES, 1 µg/ml TPCK-trypsin and 0.8% Avicel for H1N1. Infected cells were fixed and stained with 4% paraformaldehyde and 0.5% crystal violet, respectively.
